# A Biopsychosocial-Ecological Framework for Family-Framed Dementia Care

**DOI:** 10.3389/fpsyt.2021.744806

**Published:** 2021-12-17

**Authors:** Carol Ann Podgorski, Sharon D. Anderson, Jasneet Parmar

**Affiliations:** ^1^Department of Psychiatry, University of Rochester, Rochester, NY, United States; ^2^Department of Family Medicine, University of Alberta, Edmonton, AB, Canada

**Keywords:** person-centered care, biopsychosocial, social ecological, family relations, family systems, dementia care, collaborative care, caregiver-centered care

## Abstract

The biopsychosocial model has been applied through collaborative care dementia models to the diagnosis, symptom management, and treatment of dementia with a focus specifically on the person with dementia. Because individuals with dementia are increasingly dependent upon others particularly as the illness advances, dementia care requires the involvement and commitment of others, usually family, along with support from community-based resources. Hence, the quality and effectiveness of a person's dementia care are shaped in large part by the foundation of family relationships and the social and community networks in which they are embedded. While most current dementia care models incorporate biopsychosocial principles and recognize the essential role that family members play as caregivers, they fail to consider a patient's family system and relationships as potential risk factors or social determinants for care outcomes. This paper introduces a biopsychosocial-ecological framework to dementia care that is person-centered and “family-framed” in that it targets factors that influence care considerations at both the individual and relational levels of the social ecological networks that the patient and their family members occupy. We use this model to illustrate how current dementia care practices tend to focus exclusively on the individual patient and caregiver levels but fail to identify and address important relational considerations that cut across levels. We call for the need to add assessment of family relational histories of persons with dementia and family members who care for them in order to better meet the needs of the patient and the caregiver and to prevent harm. This model accentuates the need for interprofessional education on family assessments and caregiver-centered care, as well as interdisciplinary, collaborative models of dementia care that assume more accountability for meeting the needs of family caregivers in addition to those of persons with dementia.

## Introduction

According to the World Health Organization (1) there are nearly 50 million people with dementia worldwide and projections indicate that this number could reach 82 million by 2030. The Alzheimer's Association (2) reported that over six million Americans and 747,000 Canadians (3) are living with Alzheimer's disease or a related dementia. Dementia contributes significantly to disability and dependence for older adults worldwide, and “it places a physical, psychological, social, and economic toll on those with the diagnosis as well as their caregivers, families, and societies” (1).

Manifestations of dementia extend far beyond the person with the diagnosis. Individuals with dementia are increasingly dependent upon others as the illness advances and, thus, their care needs come to require the involvement and commitment of others, usually family members. In the United States, 83% of support provided to older adults comes from family, friends, or other unpaid caregivers (2). Older adults with dementia are more likely than those without dementia to have co-morbid conditions, such as heart disease, diabetes, and kidney disease, which compounds the complexity of their care needs (2). For these reasons, the care preferences of the person with dementia must be understood in combination with the preferences of their caregivers, and within the context of their family relationships and the social ecological contexts in which they are embedded. In 2021, the Alzheimer's Association (2) estimated the cost of caring for those with dementia to be $355 billion, including $239 billion in combined Medicare and Medicaid payments, in addition to the estimated $257 billion worth of care provided by family and unpaid caregivers.

Nearly three-quarters of those providing care to someone with dementia, one-third of whom are 65 years of age and older, expressed concern about maintaining their own health since taking on a caregiving role (2). Over ~30–40% of family caregivers report depression and 44% report anxiety (4). The ongoing stress of caregiving has also been linked to impaired sleep, increased hypertension, impaired immune function, slowed wound healing, and increased inflammation (2). While employment can be a relief, that is it can counter-balance caregiving strain at home, caregiver in Canada and the United States experience more caregiving-work conflicts and tend to reduce work hours or stop working as the dementia progresses (5–7). Financial strain adds to caregiver stress (8, 9). The most recent American Association of Retired Persons study found that on average family caregivers are spending 26% of their income on caregiving activities (9). Because the responsibility for providing care to someone with dementia places such a toll on those who do, an integral part of dementia care involves supporting and sustaining caregivers. With families providing the majority of dementia care, supporting caregivers has become a public health priority (10, 11).

Person-centered care has been widely adopted as the gold standard of care for older adults, including those with a dementia diagnosis (12, 13). As defined by the Institute of Medicine (14), person-centered care is that which “is respectful of and responsive to individual patient preferences, needs, and values and ensuring that patient values guide all clinical decisions.” The provision of person-centered care to those with dementia becomes complicated, however, as the person's dependence on family and friends increases to the point at which the needs and preferences of the person(s) providing care must also be considered in care planning. In an effort to call attention to the needs of family caregivers, Parmar et al. from Alberta, Canada (15–17), developed a comprehensive set of caregiver-centered competencies aimed at training healthcare professionals to better recognize and address the needs of caregivers. They coined the term “care giver-centered” to specifically focus on a person- and family-centered approach to supporting caregivers as well as the people they care for. When the needs and preferences of more than one person are involved in a care decision relationship factors among those involved become an essential consideration in the care planning process. It was the co-authors' mutual recognition of the narrow lens through which caregiver needs are commonly addressed in dementia care settings, in both the United States and Canada, that brought us together as collaborators on this project.

In this paper we propose a conceptual framework that applies principles of both the biopsychosocial and social ecological models to person-centered care for the person with dementia and the family member(s) who care for them as a way to illustrate the significant influence that family relational factors have on a patient's and caregiver's experience of dementia.

## History of the Biopsychosocial Model and Dementia Care

Engel (18) described the biopsychosocial (BPS) framework as “a scientific model constructed to take into account the missing dimensions of the biomedical model.” Originally proposed as a framework to shape diagnostic and treatment approaches to psychosomatic illness (19), Engel called on physicians to attend to the ways in which biology, psychology, and social issues contribute to the presentation of health and treatment response at the psychological, physical, and social levels of functioning (20). Since the seminal publication in 1977 (19), the biopsychosocial model has become an accepted clinical paradigm not only for medical education but also for professions including nursing, social work, psychology, and marriage and family therapy, and it is also the foundation upon which medical family therapy and integrated care were developed (21). The BPS has been applied to the art and science of medicine, to patient and physician experiences, and to myriad physical and mental illnesses.

There are few published reports on applications of the biopsychosocial model to dementia. In the United Kingdom, the biopsychosocial model was used by the National Institute for Health and Clinical Excellence and the Social Care Institute for Excellence (22) to inform the development of guidelines for clinical practice and evidence-based decision-making related to dementia care. Keady et al. (23) described how these guidelines aligned the biological, psychological, and social domains to manifestations of dementia symptoms but failed to address the physical symptoms and, hence, proposed the utility of a bio-psycho-social-physical model of dementia. Spector and Orrell (24) proposed a working BPS model of dementia focused on the patient's needs over the course of the illness. They differentiated between the biological and psychosocial factors that are fixed and those that are tractable in an effort to inform intervention strategies. In these three BPS applications the focus of care was limited to the needs of the person with dementia.

In addition to clinical applications, the BPS approach has also been applied toward understanding variations in family awareness of Alzheimer's disease during the pre-diagnostic phase of cognitive impairment. Clare (25) published a review of the models that explained variations in awareness of observable changes in people with early-stage Alzheimer's disease. The author concluded that understanding variations in awareness requires a BPS model of awareness that takes into account neuropsychological impairments and psychosocial responses by others, and that understanding both was essential for developing person-centered dementia care. Clare et al. (26) later tested a BPS model of awareness in early-stage dementia by gathering evidence regarding the relative contributions of neuropsychological, individual psychological, and social factors to measures of awareness. Their findings supported use of a BPS framework in that psychological and social factors, along with illness-related and neuropsychological factors, were found to significantly influence the degree of awareness. In a related effort Rogers et al. (27) conducted a review synthesizing qualitative research exploring family members' experiences of the pre-diagnostic phase of dementia to inform clinical practice. They found that family members engage in a “sense-making” process throughout the pre-diagnostic period. In line with findings by Clare et al. (26) they reported that families made sense of the changes they saw in the affected family member by observing, appraising, and reacting to changes and that the social network influenced their appraisals and responses to change. This set of papers illustrates the important role that families play in determining the timing of diagnosis and in shaping the narrative that ultimately informs the history of presenting illness at the point of diagnosis.

## Beyond Biopsychosocial: Adding a Social Ecological Perspective to Dementia Care

Engel (18) acknowledged the existence of two hierarchies in that “the single individual (person) is the highest level of the organismic hierarchy and at the same time the lowest unit of the social hierarchy.” He also noted (18) that be it a cell or a person “nothing exists in isolation,” and every system is influenced by the environment or “configuration of systems” of which it is a part. Herein lies our rationale for adding an ecological component as a necessary extension of the BPS approach in dementia care. In what follows, we begin by describing how Engel's model falls short in addressing the needs of the person in the context of their lived experience of dementia. Then we move on to illustrate how the social ecological model allows us to better understand the person with dementia within their family relationships and social networks, which helps to capture a more comprehensive picture of the person's individual and relational needs regarding dementia care. Then, acknowledging that dementia care is shaped by relational factors, and incorporating the caregiver-centered work of Parmar et al. (15–17), we call attention to the need for dementia care models to go beyond the BPS and into the relational level of the social ecological model of the family member(s) who provide care.

Around the time Engel introduced the BPS model, Bronfenbrenner (28) published his groundbreaking work, *The Ecology of Human Development*, the premise of which is that human development is shaped by the interaction between an individual and their environment. This was the genesis of ecological systems theory and the social ecological model (SEM) that continue to be applied to understanding a host of social issues, including many related to public health and social determinants of health.

There are few publications focused explicitly on the integration of the BPS and social ecological frameworks. One such effort in health psychology integrated concepts from the BPS and ecosystemic models, including the SEM (29). They developed a “dynamic model of health” to explain the interactive elements of the BPS model and the social ecological approach to elaborate interpersonal dynamics within social environments that modulate influences on health. This study lends credence to our rationale for incorporating these two models to better capture relational elements that are currently missing in dementia care models.

Other applications of the SEM to dementia include the following. The Changing the Person, Changing the People, and Changing the Place Model developed training for caregivers to promote maximal independence in individuals with dementia during meal time (30). Cho et al. (31) used data from the multisite Resources for Enhancing Alzheimer's Caregiver Health II (REACH II) intervention and applied a socioecological framework to determine the extent to which intrapersonal factors, intrapersonal processes and groups, and organizational factors could constrain or promote individual behaviors to influence the “positive aspects of caregiving.” O'Shea et al. (32) used a social ecological framework to understand how various stakeholders perceived access to respite services and to explore the boundaries of public responsibility in relation to client care preferences. Wang et al. (33) used a social ecological approach to understand how individual, interpersonal, and community level factors influenced informal caregiver appraisals of their caregiving experiences.

Ecological systems theory has also been applied to the lived experience of people with dementia as addressed in this set of studies. Clarke et al. (34) found that by addressing individual and community needs, communities could develop services that promoted independence, control and choice, and enable people to re-narrate their lived experiences within their communities as purposeful. Gorska et al. (35) examined the emerging experience of people living with dementia and found that their potential to adjust to continuous changes is influenced by access to and quality of both personal and contextual resources which remain in a constant, transactional relationship to each other. They later found the process of adaptation to be one that involved active participation through ongoing, dynamic and non-linear interactions between the adaptive capacity of a person with dementia and the adaptive capacity within the environment (36). Together these studies indicate the importance and value of considering people with dementia and their needs within the relationships and contexts of their larger environments.

## Current Models of Dementia Care: Biopsychosocial and Social Ecological Considerations

There are many published reports of effective, evidence-based dementia care models (37–42), all of which are interdisciplinary, collaborative, and address the BPS needs of patients with dementia. They are based in primary care, in geriatrics, or in specialty care practices; some have a home-based component; others are co-management models with primary care; all have case management services and collaborate with community providers; and some include palliative care. These models focus on providing accessible, person-centered, and socioculturally appropriate care, while improving health outcomes and reducing costs (41, 42). By acknowledging the complex interaction of cognitive, functional, behavioral, and psychological symptoms that contribute to decreased quality of life for the person with dementia and family caregiver(s), they largely call for health care professionals to address the BPS needs of the patient along with the caregiver's needs for dementia education and support. Boustani et al. (37) from the Healthy Aging Brain Center in Indianapolis, describe the need for care models that improve health outcomes for patient and caregivers through pharmacological and non-pharmacological interventions “specific to the dementia-related disability.” From our lens we perceive limiting the scope of assessment and intervention to “the dementia-related disability” as shortsighted in adequately addressing the course of a disease that is shaped so significantly by relational and ecological factors.

In most dementia care models the focus on the caregiver is limited largely to bolstering the caregiver with the goal of sustaining care for the person with dementia. The caregiver is viewed less often as a person with needs of their own or as a partner on the care team (43). More recently dementia care investigators have recommended that models specifically assess and address caregiver's support needs to better assist them in their caregiving role and to maintain their well-being. Queluz et al. (44) published results from a scoping review of 31 studies on needs of dementia caregivers. Choosing from among fixed-choice options, personal health (58% emotional health; 32% physical health) and receiving help from others (55%) were the most frequently endorsed caregiver needs. Queluz et al. (44) noted, however, that the investigators' concluding recommendations did not address the two most commonly cited needs but, rather, focused on information gaps and education needs of caregivers, the two needs most routinely addressed in clinical practice.

Leading dementia models use comprehensive assessments comprised of validated measures to guide care planning, including support for caregivers. Investigators of the MIND at Home program at Johns Hopkins University conducted a randomized controlled trial to test an intervention designed to systematically identify and address dementia-related care needs through individualized care planning, referral and linkages to services, provision of dementia education and skill-building strategies for caregivers, and care monitoring by an interdisciplinary team (38). The domains of need they assessed included home and personal safety, general health care, daily activities, neuropsychiatric symptoms and legal concerns. At baseline the most frequently addressed unmet needs of those with dementia included personal and home safety, general health and medical care, meaningful activities, legal and advance care planning, and diagnosis of dementia. Caregivers most often received referrals for resources and education. The UCLA Alzheimer's and Dementia Care program also administers a comprehensive assessment of patient and caregiver needs that address domains that capture BPS needs of patients, and caregiver issues including caregiver stain, depression, and needs for services.^1^ At the outset of the program referrals were most commonly made for support groups, wandering support, caregiver training, and medication adjustment (39). Neither of these programs assesses for relational histories or family factors that could influence the care plan or quality of life for the person with dementia or their caregiver(s).

In addition to addressing BPS concerns of the patient, the Healthy Aging Brain Care program also assesses caregiver needs, including depression, strain, burden, and physical and emotional strain. While this assessment does not address the quality of family relationships, it does address factors within the caregiver's social ecological system by addressing their living situation, other caregiving and competing responsibilities, worries, and sources of support.^2^

While these programs are all highly attuned to the dementia patient's needs and to the needs of the family member as caregiver, none addresses the quality of the relationships between the patient and the family caregiver(s), how they function as a family, or any past or present interpersonal safety or trauma-related concerns that could affect care plan implementation, quality of life, or health outcomes. In addition, following assessment, caregivers are often referred to other community resources to get their needs met and it is unclear whether there is coordination or communication back to the dementia care program regarding the outcome of those referrals. As Aldridge et al. (45) contend, each service often serves the family “in isolation” from each other as opposed to working collaboratively. They conclude that this often leads to a poor understanding of each other's roles in supporting the “collective complex needs of the family.” Further, O'Shea et al. (32) described this type of care as being embedded in a system “configured to deliver a biomedical model of care and which assumes non-medical care is a family responsibility.” While these models are all effective in doing what they are designed to do, the designs do not address understanding the needs of those with dementia and their family caregivers in a way that acknowledges the power of family relational dynamics in shaping the dementia or caregiving experience, clinical encounters, or outcomes of care.

## A Biopsychosocial-Ecological, Family-Framed Approach

Lyman Wynne, MD, PhD [(46), pp. 220–221] in reflections on conversations he had with his longtime colleague George Engel pointed out that Engel was “clear and explicit” in recognizing ways in which the system levels were different yet linked [(46), p. 221]. Engel (18) stated, “Each system implies qualities and relationships distinctive for that level of organization” and further argued that “in no way can the methods and rules appropriate for the study and understanding of the cell as cell be applied to the study of the family as family” [(46), p. 221;12]. Similarly, in no way can the methods for assessing dementia-related BPS factors of those with dementia capture the essence of the relationships and communities in which they are a part; nor can they capture the individual, relational, or social factors that influence the family member's performance as caregiver. Thus, this is our rationale for focusing on the ways in which interactions within and between the individual and relational levels of a person's social network influence the experience of dementia for the person with dementia and the family caregiver(s).

Family relational factors and dementia care outcomes have been examined. Relationship satisfaction prior to diagnosis was found to be negatively associated with caregiver burden in that caregivers with high satisfaction reported less burden and reactivity to memory and behavior problems, and better problem solving and communication skills (47). Caregivers reporting poor family functioning at time of diagnosis expressed higher ratings of strain and burden (48). Increased caregiver burden and strain were related to poor emotional responsiveness, problem solving, and communication (47) and to impairment in role functioning and emotional involvement (47, 49). Decades of research show family relational factors that adversely affect health include: high interpersonal conflict, low relationship satisfaction, poor problem solving skills, high levels of criticism and blame, intra-family hostility, poor family organization, inconsistent family structure, family perfectionism and rigidity, low family cohesion, lack of closeness, and lack of an extra-familial support system [(50), p. 204]. Protective factors include: good communication, adaptability, clear rules, mutual support, open expression of appreciation, commitment to the family, spending time together, good problem solving skills, and an extra-familial support system [(50), p. 205]. Yet family relationships are largely ignored in clinical settings.

## Case Presentation

The following case is presented to illustrate, using a biopsychosocial-ecological perspective, three different approaches to serving persons with dementia and their caregiver(s) in clinical practice.

**Presenting concerns**: Janice is an 85-year-old woman who lives independently in senior housing in the Canadian province of Alberta. In response to Janice's increasing needs for support, Gwen, her daughter and primary caregiver, scheduled an appointment for them to meet with her mother's Geriatrician to discuss changes in Janice's health and function related to her progressing dementia, and planned to discuss her own needs for support as well.

Gwen reported to the geriatrician that her mother's decline had been steady since her last appointment, most notably in her short term memory such that she was increasingly losing items, struggling to recall recent events, forgetting names, and having difficulty finding words, managing complex tasks, and planning. She shared that her mother had developed paranoia and visual hallucinations over the past year during which she imagines that strangers are trying to get into her home to steal her treasured belongings. The hallucinations had increased steadily and had worsened over the past month now occurring multiple times per week usually at night. Gwen also reported that Janice calls her frequently asking for help, and she noticed her mother being more irritable, angry, and frustrated than she used to be. She shared that her mother wanders out of her room but has not gotten lost.

Gwen also noted a “quite rapid” decline in Janice's function. Because she was no longer able to use the stove and had burned pots, she ultimately stopped cooking and depends on microwave-ready meals and easy snacks. Even with Gwen bringing her meals, however, Janice has had a 20 pound weight loss over the past year. Janice can still perform basic activities of daily living such as dressing, grooming, bathing, feeding, toileting, transfers and mobilization. She can still use the phone and does housekeeping and laundry on her own, but Gwen finds clothes soaked in urine in the laundry and believes that her mother has not bathed in a month. Gwen now manages her mother's money, medical appointments, and medications, and does her shopping and other errands as well.

Janice's neighbors and building management started to raise concerns to Gwen about her mother's safety, which Gwen reported has greatly increased her own anxiety about her mother's living situation. They reported that Janice is seen wandering around the facility at all hours and often checks in with other residents when she gets confused about day and time. There are times when she will knock on her neighbors' doors asking for help while experiencing hallucinations. They know her well and reassure and redirect her but Gwen wonders how long they will be willing to do this. Janice adamantly denies needing assistance but Gwen was finally able to get her to accept homecare for help with medications. The agency recently informed Gwen, however, that Janice does not always open the door for the homecare attendants and that she sometimes calls them derogatory names and yells at them to “get out.”

**Concurrent problems**: While Janice has experienced urinary incontinence for years, she was managing on her own with pads and then protective underwear as the incontinence worsened. Gwen describes her mother's bladder control as “good during the day” but notes that she “occasionally soaks her night clothes and bed during the night.” Janice also has occasional bowel incontinence and Gwen noticed that her pericare had declined and shared that she had found smeared stool around the toilet. The geriatrician also expressed concern about Janice's sensory deprivation noting that she is legally blind due to macular degeneration and that she suffers from bilateral hearing loss and has been unable to manage hearing aids on her own. Janice's other medical conditions include hypertension, osteoporosis, osteoarthritis, and hypothyroidism. She never smoked, rarely consumes alcohol, and gave up driving 3 years ago because of her vision loss.

**Mental exam**: The geriatrician noted that Janice was alert and cooperative and that she needed a pocket talker to hear. She scored 24/30 on the Mini-Mental State Exam (51) and 18/30 on the Montreal Cognitive Assessment (52), both of which indicate “mild dementia.” The Clock Drawing Test (53), a measure of spatial dysfunction and neglect, was abnormal. She correctly placed the numbers on the clock face but could not tell time. She had problems with orientation and displayed both short and long term memory deficits. Language skills were intact other than occasional word finding problems. She appeared anxious and got easily irritated. She needed reassurance to complete the assessment. She was occasionally distracted by visual hallucinations (e.g., she saw people in the room and wanted them chased away). She denied symptoms of depression. She had poor insight into her cognitive and functional decline and displayed poor mental reasoning when it came to supports needed to help her with her health and housing. She overestimated her abilities and did not recognize the degree of supports being provided to her. She acknowledged that her daughter provides some help but said she could manage without it. She expressed annoyance with having homecare.

**Physical exam**: No apparent distress.

**Family and social history**: Janice completed education through Grade 8 and worked as a secretary until she had children. She has been widowed for 20 years after having been a caregiver to her husband who died of cancer. She has 3 daughters, 1 son, and 8 grandchildren. Gwen, the youngest, her primary caregiver, and “the baby” of the family, is married, has 2 children, and lives 10 min away. Janice's son, Jack, is an accountant who lives out of town, helps with higher level financial management such as taxes, and is a source of emotional support for Gwen. Janice often mentions that Jack “leads a busy life with work and family” as an explanation for his infrequent visits. Her two older daughters are both married, retired, and live in other provinces. They check in about their mother periodically and visit once a year. Neither of the two older daughters is close to Janice or Gwen with the emotional distance rooted in their shared belief that their mother favored their two younger siblings when they were growing up. Gwen and Jack have remained close and frequently discuss their mother's deteriorating health and function. Janice has lived in her current residence, a subsidized senior housing facility, for the past 30 years. She has limited finances, including her husband's pension and her own, and she relies on her children to assist with money as needed.

**Patient's values and beliefs**: Janice does not want to leave her home. She is feisty and wishes to remain independent. She is fond of her belongings and takes pride in them– e.g., furniture, paintings, pictures, collectibles, etc. She believes that she raised her children well and gave them a good education, and she now expects reciprocity. She acknowledges the support provided by her daughter but is not particularly empathic toward her stress.

**Medical and legal issues**: Janice designated Gwen and Jack as the agents in her Personal Directives and Enduring Power of Attorney (EPOA), respectively. The EPOA was activated at the time it was established. Janice's Goals of Care Designation, a medical order used in Alberta to describe and communicate the general focus of care including the preferred care location, indicates that goals and interventions are for cure or control of illness. Her goals exclude the option of ICU care, while transfer to an acute care facility may be considered if required for diagnosis and treatment.

**Caregiver stress**: Gwen is committed to caring for her mother and determined to support her at home. She reported that she had promised not to relocate her to a “nursing home.” However, she admits to feeling “very stressed” caring for her mother. She is the only one in town and has taken over the majority of the responsibilities. Janice is quite demanding and calls her day and night asking for help. She gets easily irritated and angry with Gwen who has already reduced her hours at work by going part-time. Gwen believes at this rate she will have to quit work all together. This adds to her stress because she feels guilty about harming her family's financial situation. She and her husband annually spend $6,000 subsidizing her mother's housing, food, and health care supplies. Gwen is keenly aware that their daughters are approaching college age and that this is not the time to leave the workforce. She feels that her life is “on hold.” Her husband and children are supportive and help however they are able. She resents the lack of support from her sisters but finds her brother more supportive as he provides her with emotional support and helps to support their mother financially. At the same time she feels he could visit more often. She shared that caregiving is taking a toll on her health as she is experiencing panic attacks, insomnia, poor concentration, feelings of guilt, and chronic migraines, in addition to having emotional and physical symptoms associated with perimenopause.

## Impression and Interventions

***Patient-centered***: Janice meets criteria for Mixed Dementia (Major Neurocognitive Disorder) with Behavioral Psychological Symptoms of Dementia, with impairments in memory, insight, judgment and executive function. The neurobehavioral issues include easily irritability and anger, verbal abuse and hallucinations of a persecutory nature. The sensory deprivation due to macular degeneration and hearing loss could be playing a role.

Her dementia is approaching moderate severity with a loss of function primarily in instrumental activities of daily living. Her function could be maintained with increased homecare and support from her daughter. She needs monitoring of medications, caloric intake, and weight and needs to be encouraged to drink fluids as she is at risk for malnutrition and dehydration. She also needs reminders to take a shower and tend to periodic pericare. Her refusal of homecare is problematic. The Geriatrician reviewed the options with her daughter, including self-managed care and having a consistent care provider and overnight care. Gwen agreed to install a locked box to give access to the homecare attendants who will also assist her hearing aid use. The case manager has good rapport with Janice and will work with her to accept help. The hallucinations need aggressive treatment because the patient is experiencing them frequently and acting on them. Increased Quetiapine to 50 mg qhs and 12.5 mg q6h prn. Homecare attendants and her daughter will monitor for side effects. Bloodwork ordered through home collections to rule out anything acute.

Janice lacks capacity to make decisions in the domains of health and accommodation, and is making decisions that are putting her in harm's way. The Personal Directive needs to be enacted which will give Gwen the authority to make decisions for her in these two domains.

***Caregiver-centered***: The Geriatrician also addressed Gwen's stressors. Following at-length discussion Gwen agreed to referrals for emotional and psychological support, and for system navigation. She also agreed to contact her primary care physician to address her mental and physical health concerns. She requested a family conference with her siblings and the Geriatrician asked the case manager to arrange one to discuss the possibility of more family cohesiveness in providing for Janice's care and decision-making. Gwen also expressed an interest in learning to set limits with her mother and agreed to a social work referral to discuss strategies. She acknowledged that enacting the Personal Directive may increase her sense of control.

***Goal***: ***To support Janice in her current residence***: Based on previous conversations and verified again at this appointment, the Geriatrician ascertained that Janice's strong preference is to remain in her current residence. Janice expressed that her greatest fear is being evicted and that she “wants to stay there at all costs.” Her daughter acknowledged that she would like to honor her mother's wishes.

## Viewing the Case through the Biopsychosocial-Ecological, Family-Framed Lens

**Figure 1:**
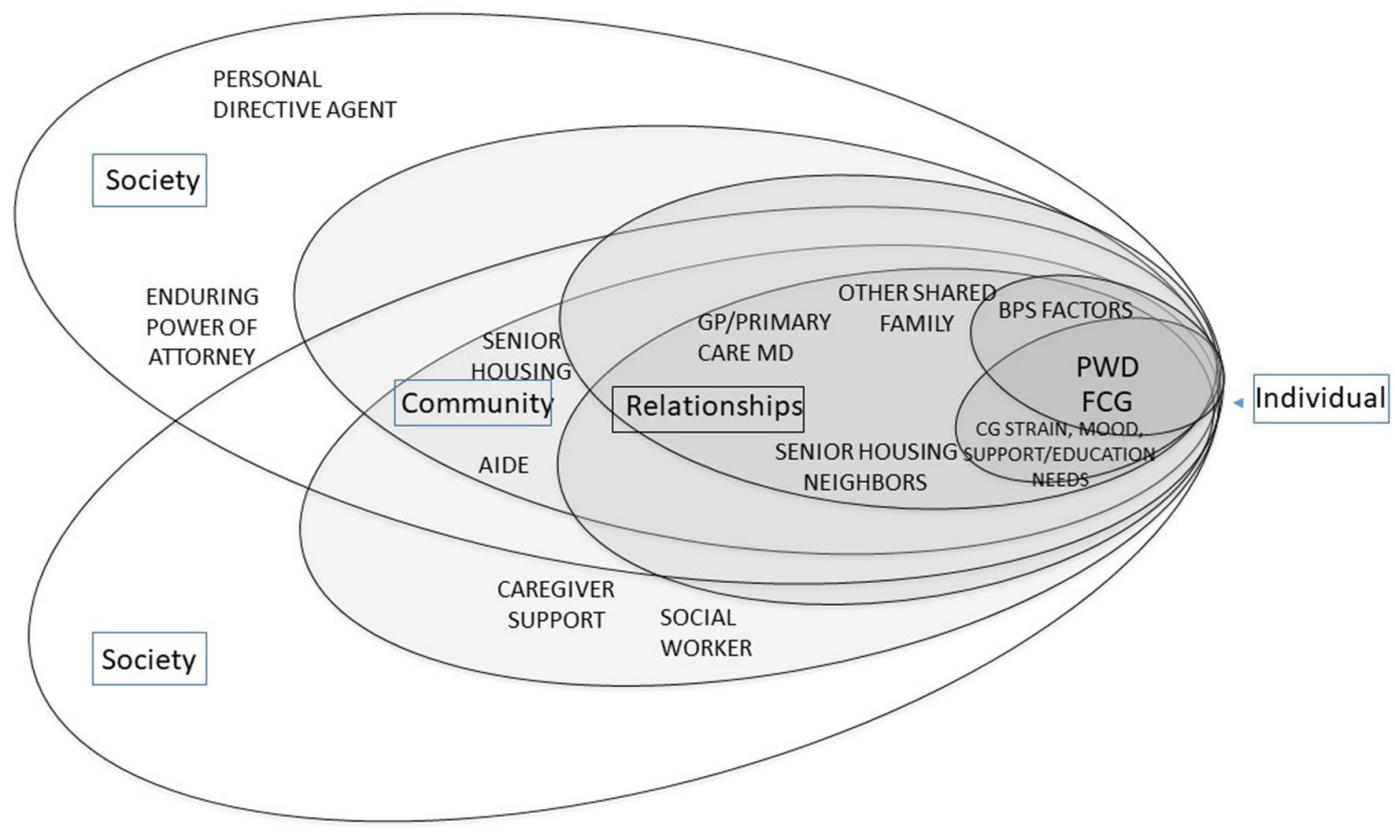
Biopsychosocial-Ecological Approach to Patient-Centered Dementia Care.

The biopsychosocial and social ecological factors associated with Janice and Gwen, as presented in the case, are delineated in Tables 1A,B, respectively. Figure 1 illustrates how most dementia care models, based upon what they assess as described earlier, view the needs of the person with dementia (PWD) and their family caregivers (FCG). By and large they address the BPS needs of the PWD relative to dementia, and consider the FCG's needs in relation to maximizing support of the PWD's plan of care by addressing needs for dementia-related education and support.

**Table 1A:** Examples of biopsychosocial and social ecological considerations for a person with dementia: the case of Janice.

**Identifying Information: Janice** is an 85-year-old woman with advancing dementia who lives in Alberta, Canada		
**Reason for Assessment:** Dementia requiring support for care planning and medical intervention		
**Dementia Diagnosis:** Mixed Dementia (Major Neurocognitive Disorder) with Behavioral Psychological Symptoms of Dementia		
**Biological/functional**	**Psychological/behavioral**	**Social**
**BIOPSYCHOSOCIAL CONSIDERATIONS**		
• Steady decline in cognition >2–3 years • She scored 24/30 on the MMSE and 18/30 on the MOCA; abnormal Clock Draw Test; observed impairments in memory, insight, judgment and executive function • Medical conditions: hypertension, osteoporosis, osteoarthritis, hypothyroidism • Sensory deprivation: legally blind due to macular degeneration; hearing loss with declining ability to use hearing aids • Urinary (urge) incontinence, worse at night; bowel incontinence, occasional • Steady, now rapid, functional decline >1 year; can manage dressing, feeding, transfers, and mobilization; requiring assistance with toileting, bathing, grooming	• Experiencing episodes of paranoia • Experiencing persecutory visual hallucinations >1 year, increasing in severity • Has poor insight regarding her cognitive and functional abilities; overestimates her abilities and does not recognize the degree of supports being provided to her • Becomes irritable easily and expresses anger and frustration regularly • Consistently refuses idea of homecare • Refuses to let attendants into her home at times • Wanders around housing complex	• Was born and raised in Canada • Completed Grade 8 education • Worked as a secretary before children • Was married and has 3 daughters, 1 son, and 8 grandchildren • Widowed for 20 years; had cared for her husband who died from cancer • Has lived independently in subsidized housing for 30 years; has limited finances • Seeks support from neighbors • Can no longer use stove. Relies on microwave-ready meals and snacks • Can no longer manage finances, medical appointments, medications, shopping • Stopped driving 3 years ago (vision loss) • Safety risks with stove and wandering • Hygiene concerns (e.g., smeared feces around toilet, urine-soaked clothing in laundry) • Has medication management assistance
**Individual level**	**Relational level**	
**SOCIAL ECOLOGICAL CONSIDERATIONS**		
• Goals of care per patient's documented wishes: Supportive care, symptom management and comfort measures only • Patient does not want to leave her home • Is strong-willed and values independence • Cherishes and takes pride in her home of 30 years (i.e., her decorations, furniture, paintings, pictures, collectibles etc.) • Believes that she did well raising her children and giving them a good education and expects reciprocity • Overestimates her abilities and does not recognize the degree of supports being provided to her • Lacks capacity/Personal Directive regarding domains of health and accommodation to be activated		• Janice has 3 daughters, 1 son, 8 grandchildren • Youngest daughter, Gwen, is her primary caregiver who lives 10 min away. She is married, has 2 children, and has reduced her work hours to accommodate her mother's needs • Patient's son, Jack, is an accountant, lives in another province and helps with higher level money management such as taxes. He leads a busy life with work and family. • Janice's 2 older daughters, also married, live in other provinces. Both retired they check in with caregiving daughter periodically about their mother and visit her once a year. They are not close to their mother or sister as they felt their mother favored the their two younger siblings when they were growing up • Gwen and Jack have remained close and discuss the issues and care plans around their mother's deteriorating health and function • Gwen is the agent in her Personal Directive (i.e., Advance Directive) and Jack is agent for the Enduring Power of Attorney (EPOA), which became effective on the date it was established • When the Personal Directive was recently enacted Gwen was granted authority to make decisions for her mother regarding health and accommodation (housing) • Janice checks in with other residents around day, time etc. She will often knock on her neighbors' doors asking for help with her hallucinations. They know her well and reassure and redirect her • Janice and Gwen have good relationships with patient's primary care physician, a geriatrician	

**Table 1B:** Examples of biopsychosocial and social ecological considerations for a family caregiver: the case of Gwen.

**Identifying Information: Gwen** is a married 45 year old woman with two children, a job, and responsibilities as primary caregiver for her 85 year old mother with advanced dementia who lives nearby in Canada		
**Reason for Assessment:** Caregiver stress		
**Biological/functional**	**Psychological/behavioral**	**Social**
**BIOPSYCHOSOCIAL CONSIDERATIONS**		
• Symptoms of depression (e.g., difficulty falling and staying asleep, low energy, poor concentration, feelings of guilt and failure) increasing in frequency and intensity since her mother's care needs increased • Symptoms of anxiety (e.g., fatigue, poor concentration, heart palpitations, occasional panic attacks) increased as concern for her mother's safety arose • Medical conditions: hypertension, chronic migraines, perimenopause	• Has been taking more sick days off from work and worries about losing her job • Stopped playing in the volleyball league she and her husband always enjoyed and looked forward to each summer due to fatigue and headaches • Relies on her two high school aged children to take care of things at home while she tends to her mother's needs • Formerly a proud multitasker, she now focuses only on one thing at a time and gets anxious when asked to change her focus without notice • Feels disconnected from her husband and children and they feel disconnected from her too • Has a hard time accepting help from friends • Lives with anxiety, sadness, and guilt that her mother's condition will deteriorate and that she will have to make the decision to move Janice from the home she cherishes to a facility with more care. • Fears losing her job and related income	• Was born and raised in two-parent household in Canada • Married for 20 years, two children • Employed as a dental hygienist part time • Husband travels occasionally for work • Has a small, close circle of friends
**Individual**	**Relationships**	
**SOCIAL ECOLOGICAL CONSIDERATIONS**		
• Mother's geriatrician set goal to reduce caregiver's stress and refers her to meet with a social worker to learn about care options for her mother • Caregiver “misses her former self and her family” • Husband encourages caregiver to see her primary care physician and offers to attend visit with her • Primary care physician's goals for Gwen are to reduce symptoms of anxiety and guilt, reduce the frequency and severity of her migraines, and manage physical and psychological symptoms associated with perimenopause. Recommends medications and provides a referral to a behavioral health specialist for psychotherapy	• Primary caregiver for her mother with whom she is very close. • Is married, has 2 teenage children, and has reduced her work hours to accommodate her mother's needs • Close relationship with her brother who is an accountant, lives in another province and helps their motherwith higher level money management such as taxes. The two are close and discuss plans regarding their mother's deteriorating health. He has limited ability to provide in-person support for their mother. • Distant relationships with two older sisters, also married, who live in other provinces and visit their mother once a year. • She is the agent for mother's advanced directive (i.e., Personal Directive) and her brother is agent for the Enduring Power of Attorney (EPOA), which is in effect • She was recently granted authority through her mother's Personal Directive to make decisions for her mother regarding health and accommodation (housing) • Has a good relationship and relies on her mother's geriatrician and social worker for support and guidance • Is reluctant to seek mental health care because it is “one more thing to do” and will “take a lot of energy” • Her husband and children express relief and hope when she decides to seek care for herself and realign her priorities • Employer supports short term medical leave from job to restore her health; caregiver feels relief for time to focus on her health and grateful for her colleagues' willingness to cover in her absence	

**Figure 2:**
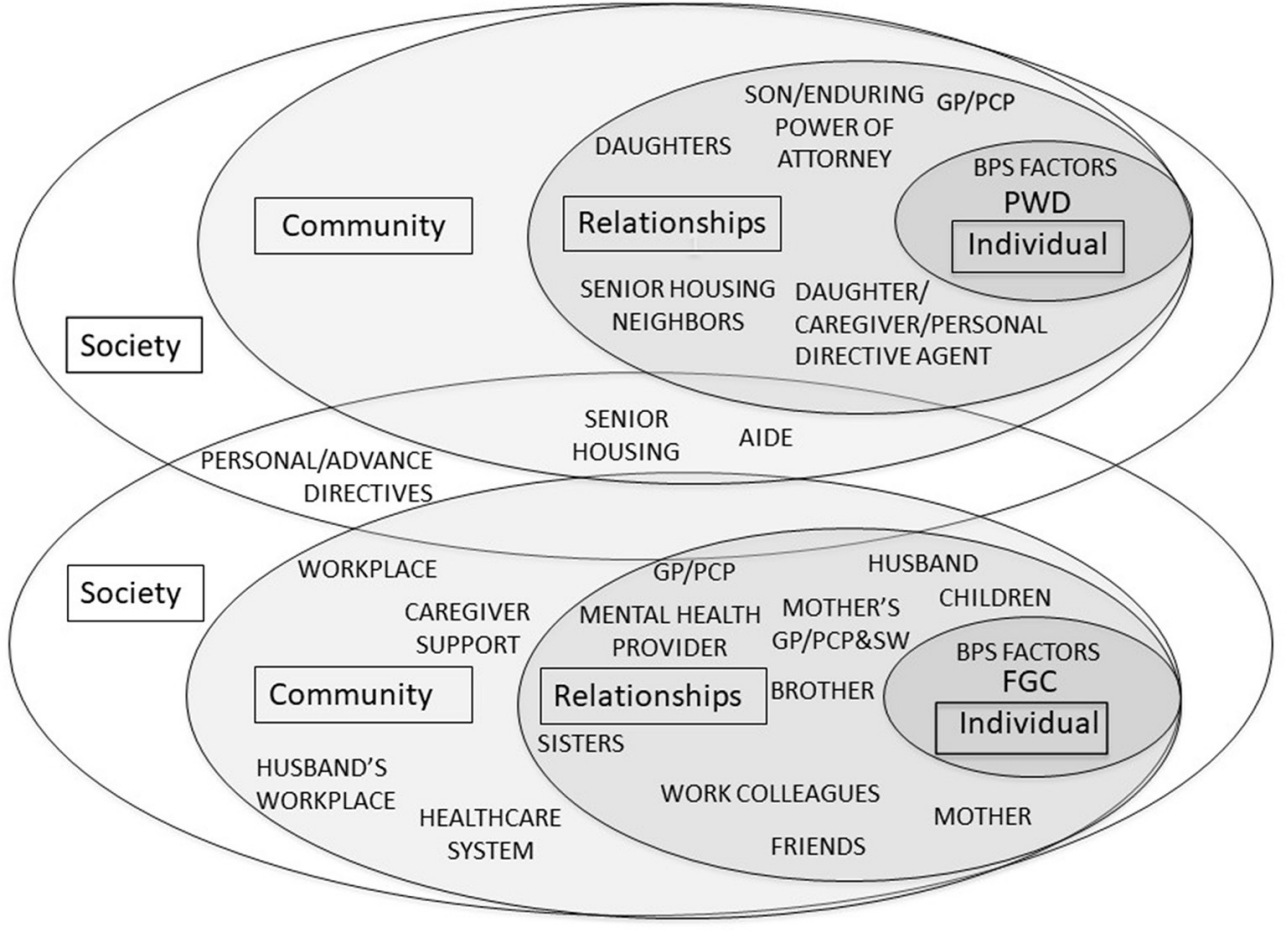
Biopsychosocial-Ecological Approach to Patient-Centered and Caregiver-Centered Dementia Care.

Figure 2 illustrates a model that acknowledges that the PWD (Janice) and FCG (Gwen) occupy distinct social ecological systems albeit with minimal overlap in this case. The caregiver-centered competencies developed by Parmar and colleagues (9–11) reflect this understanding that one cannot know best to support a caregiver by assessing the person with dementia. In this figure the BPS factors are included within the individual levels for Janice and for Gwen. For Janice, her Geriatrician, daughter Gwen, and son Jack plan for her care and increase services as needed to allow her to remain in her home as she wishes. Neighbors in the housing complex redirect her as needed. Her other daughters visit occasionally. This figure also elucidates the contextual factors which facilitate and impede Gwen's role responsibilities and well-being. It shows the resources and relationships she has available within her social network—i.e., husband, children, health care professionals, friends, aging service providers and support networks, friends, and colleagues. While this figure identifies the factors that likely influence the experiences of dementia for both Janice and Gwen, it does not offer much in terms of the ways in which the interpersonal relationships or family dynamics affect the well-being of either or their experience of dementia. This figure is akin to looking at a family photo album to see who is in the family but without access to a companion journal with detailed accounts of the family history or relational dynamics that shape the way its members function and relate to one another today.

**Figure 3:**
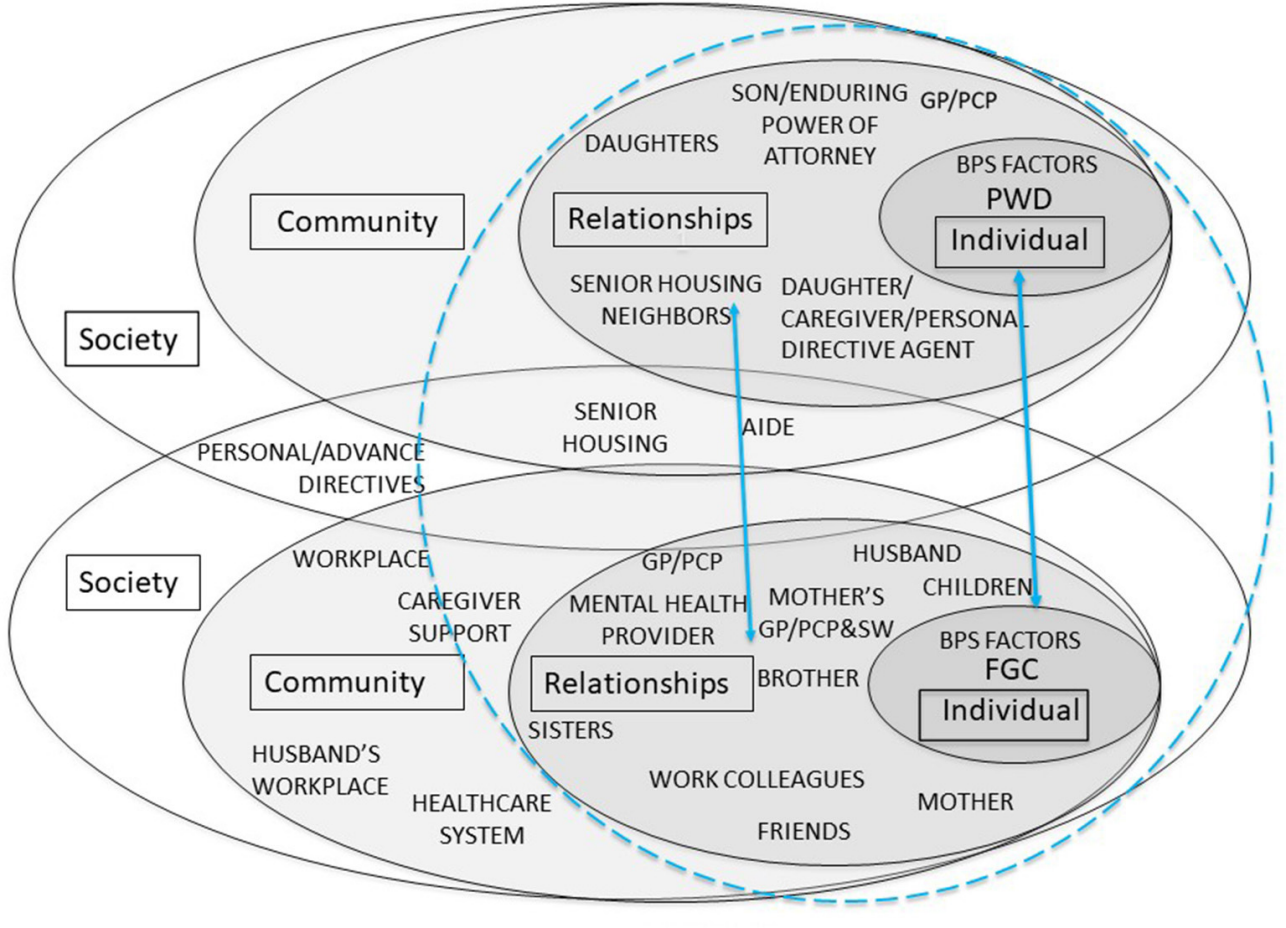
Biopsychosocial-Ecological Family-Framed Approach to Dementia Care.

Figure 3 identifies the “family-framed” domain of the biopsychosocial-ecological model that is generally missing from current dementia care practices. This figure illustrates that the PWD and the FCG each occupy a separate socioecological system, as in Figure 2, but also reflects the need to understand the important relationships for each individual as well as the relationship dynamics between the PWD and FCG. The ultimate goal of family-framed dementia care is for healthcare providers to know and understand the person with dementia and family caregiver(s) within the context of their family relationships in order to develop a plan of care that meets the biopsychosocial needs and wishes of the person with dementia; and considers the needs, wishes, and resources of the family caregiver(s) so that the care plan will be feasible, likely to be implemented, and promote the safety and well-being of all involved family members. The relational context, depicted by the circle connecting the two ecosystems, represents factors including but not limited to: family of origin experiences and expectations regarding health, illness, dementia, and caregiving; relationship histories of involved family members; relationship quality and dynamics between the PWD and the FCG(s); motivations for and degree of commitment to caregiving; power dynamics and decision-making authority regarding health care and finances; and family trauma, mental health, substance misuse, and/or abuse history (54). Applying a family frame to Gwen's individual and relational considerations, one might discover that: (1) she has responsibilities for running the household while her husband travels, tends to the needs of their children, contributes needed income from her job, and serves as primary caregiver for her mother despite having her own medical and behavioral health symptoms that interfere with her ability to function as needed across various roles; (2) her family relationships have been strained by her caregiving responsibilities and she is overwhelmed by feelings of depression, anxiety, guilt, and failure; and (3) caregiver stress is not her only health concern and that those supporting her in restoring her mental health include her husband, children, her GP/PCP, her behavioral health provider, her mother's aide and neighbors, other service providers, her employer, and her work colleagues. As this set of supports is instrumental in helping Gwen, they also indirectly support Janice and her wish to remain in her home.

As in most models of dementia practice, the Geriatrician in this case is responsible for managing Janice's symptoms and care plan, and for assisting Gwen by activating Janice's Personal Directive and for helping Gwen by identifying services to meet her mother's needs for things she is unable to provide herself. Aside from referrals for caregiver support, the physician's responsibility for the caregiver's needs typically ends there. Gwen's health symptoms, however, impair her ability to provide optimal care to her mother and may impede her capacity to carry out her plan of care. While the Geriatrician is providing all of the support possible for Gwen given that she is not the patient of record, Janice's desire to remain at home is contingent upon available resources and her daughter's ability to provide or arrange for care to meet the needs that go beyond what the Geriatrician and housing facility can provide.

In our current bifurcated models of care in which the needs of the patient and caregiver are typically not addressed side by side, it is unclear, for example, who holds the responsibility to ensure that the caregiver is capable of meeting the patient's needs or if the patient's preferences are unrealistic. In most practice models the physician responsible for their patient's dementia care would likely assess for caregiver stress and then refer the daughter, as this physician did, for caregiver support, education, and assistance with navigating the social service sector. This Geriatrician, with a caregiver-centered and family-framed approach, went two steps further by enlisting the support of a case manager to arrange for a family meeting with the hope of soliciting more assistance from the patient's other children, and by obtaining social work assistance to help the daughter set appropriate limits with her mother for the sake of preserving the caregiver's health.

## Integrating Relational Care into Dementia Care: Team Training and Tools

Family caregivers often report that their needs are often overlooked in medical settings. Fisher et al. (43) reported on a symposium conducted in Canada to identify factors that affected care provision to family caregivers by healthcare professionals. A primary finding was that family caregivers require more support than they usually received from healthcare professionals. This was attributed to a number of factors, including a lack of awareness and undervaluing family caregivers; system fragmentation, engrained healthcare professional practices and attitudes; policies limiting information-sharing with family caregivers; a lack of caregiver assessments; poor communication; a lack of health workforce training regarding the delivery of emotional support to family caregivers and navigation of family dynamics; and inadequate knowledge of conditions impacting older adults.

Assessment of relational factors, while critical, constitute a time-consuming exercise that doesn't fit easily into a busy medical practice. Years ago Engel (18) pushed back against critics who argued that the BPS model increases demands on the physician and countered that “the model does not add anything to what is not already involved in patient care.” Physician perceptions of the time involved in providing comprehensive BPS care have not changed much since Engel's time (42, 55). Hinton et al. (55) reported perceptions of providers from academic medical centers, managed care, and solo private primary care practices regarding challenges managing behavioral symptoms of patients with dementia. They identified insufficient provider time, inadequate reimbursement, poor access to dementia care expertise and community resources, lack of adequate communication across the various medical, social and community dementia care providers, and the absence of an interdisciplinary dementia care team as contributing factors. The investigators concluded that “the current operational structure of primary care is not prepared to manage the biopsychosocial needs of patients suffering from dementia.” They called for more effective educational interventions for families and physicians as well as structural changes to meet the needs of patients and their families.

The efforts needed to incorporate relational care into medical practices are akin to those currently evolving to integrate assessment of social needs and social determinants of health (SDOH) into health care as a way to improve health outcomes. Healthy People 2030 (56) includes “interpersonal relationships” within the SDOH domain of “Social and Community Context” with the justification that “people's relationships and interactions with family, friends, co-workers, and community members can have a major impact on their health and well-being.” The National Academies of Sciences, Engineering and Medicine (57) released a report that investigated the feasibility of bringing social care into health care. While there is agreement on the need for healthcare to address these social factors there is no clear directive as to whose responsibility it is to carry it out or to pay for it. Implementation is further obstructed by high physician burnout rates and the fact that care and services provided by those who could support these functions are often not reimbursable (57). One small step in this direction in dementia care in the United States was legislation that enabled Medicare to reimburse physicians for care plan services that support addressing the needs of those with dementia and their family caregivers in some limited but important ways (58).

**Interprofessional training**: Efforts toward establishing a foundation for interprofessional education in dementia have been steadily increasing (41, 59–62). Some focus on the disciplines that should be involved (41), the core topics required (41), and key elements required for effective interprofessional collaboration (61). A number of programs target students across health care professions (62–64). Targeted outcomes have included improvements in student attitudes toward interprofessional education (62–64); knowledge of dementia (62, 63); collaborative interprofessional capabilities and client-centered mindset (64); and confidence (62).

Dreier et al. (41) identified the following as core topics to ensure successful interprofessional collaboration: early diagnosis; post-diagnostic support; advanced care planning for patients and caregivers; and effective collaborative care. They also proposed minimum standards for representation by discipline and recommended that team leadership and care coordination should include primary care physicians along with nurses and/or social workers. Other professions that would enhance collaborative dementia care include behavioral health providers, pharmacists, physical therapists, occupational therapists, and speech therapists. Jennings et al. (61) identified core themes for interprofessional dementia education to include: professional roles and responsibilities, with an emphasis on the post-diagnostic stage of illness; team collaboration; knowledge of dementia; and interprofessional communication skills.

The biopsychosocial-ecological family-framed approach to dementia care as proposed herein would require additional domains of interprofessional education, including a general understanding of how family systems, relationships, and dynamics affect the lived experience of dementia for those with the diagnosis and those who care for them (54); proficiency in administering a comprehensive family assessment to understand the strengths, and resources (65); knowing when to refer the patient and/or family caregiver(s) for behavioral health services or family therapy (66); and evaluating the needs of the family caregiver(s) to determine if they are willing, capable, and have the resources needed to provide the required support while maximizing their own health and well-being (17).

**Tools to support interprofessional education and relational dementia care**: Two tools were specifically developed to support interprofessional education regarding a BPS approach to dementia. The Biopsychosocial (BPS) model of dementia tool (24) was designed to encourage staff to develop personalized interventions and treatment plans for people with dementia. Revolta et al. (67) reported findings from a feasibility study addressing the impact of training staff to use the BPS model on skills, including formulation, attitude toward dementia, and sense of role competence. Similarly the Bio-Psycho-Social-Dementia-scale (68) is another validated tool appropriate for assessing family and other contextual factors that have the potential to affect care and illness experiences for patients and families. This tool was developed to rate and improve biopsychosocial functioning in dementia care, and also to facilitate interdisciplinary collaboration, promote assessment, and merge interprofessional strengths toward development of a heterogeneous team.

An essential tool to promote a biopsychosocial-ecological family-framed approach to dementia care is the shared electronic health record. Functionality that would allow community providers to which health care professionals refer those with dementia and their caregivers in support of social or relational needs would help to bridge the chasm that currently exists between health and social service providers and could potentially allow for more coordination among providers caring for both the PWD and the FCGs.

## Discussion

Family-framed dementia care calls for health care professionals in clinical settings, regardless of discipline, to meet the needs of the person with dementia and their family caregivers by understanding their needs and preferences within the context of the family structure, dynamics, and relationships. Relational dementia care is rooted in family systems theory (69) which posits that individuals cannot be understood in isolation from one another and that families are systems of interconnected and interdependent individuals. A relational approach to dementia care acknowledges that a dementia diagnosis often represents a significant life event for a family as it generates ripple effects far beyond the symptoms of the one diagnosed. Because a person with dementia will come to rely on the support of others, a care plan must address the needs and preferences of the person with the diagnosis as well as those of the family caregivers. Dementia care at the family level is relational, transactional, and often delivered in ways that reflect the nature and quality of family relationships. An awareness of relational influences help clinicians develop safe, effective, and sustainable care plans.

Family-framed, relational care does not detract from person-centeredness. Either the person with dementia, the family member, or both together can be the target of care. However, because of the relational nature of the caregiver/care-receiver relationship, there are times when shared needs would place the dyad or family at the center of care as in Figure 1. At other times, as depicted in Figure 2, their needs may be at odds. In both of these instances, addressing the needs of the dyad requires an understanding of relationships. Without doing so, the needs of one party may be inadvertently placed in opposition to those of the other.

A family-framed approach encourages clinicians to recognize that information shared by a patient or family member is frequently shaped by relational influences. In considering the role that family members generally play in dementia care—i.e., informant, interpreter, and advocate–limited awareness of family dynamics may preclude clinicians from understanding how those relationships influence not only the patient information that family members choose to share, but also how they interpret and communicate clinical information to the patient and other family members. Relational factors also influence whether and how care plans are implemented which inevitably affects patient outcomes.

This biopsychosocial-ecological model illustrates that the bifurcation of the person with dementia from the family caregiver results from chasms that exist between the biopsychosocial needs of the person with dementia and their social ecological context, and between the social ecological contexts of the person with dementia and that of the caregiver(s). In addition, the overall responsibility for the well-being of the patient and that of each caregiver are parsed across different providers in different systems that align with the biopsychosocial model (i.e., medical care, behavioral health care, and social services). The personal physician of the person with dementia and the physician for each family caregiver likely address the medical and psychosocial needs of their respective patients. They are less equipped, however, to address the relational aspects that affect the caregiving process or the health status of any of the involved parties.

Engel (18) believed that “clinical study begins at the person level and takes place within a two-person system, the doctor-patient relationship.” We contend that for dementia care it goes beyond a two-person system in that it also includes the patient's family, however defined, a relationship between the doctor and the family member(s) who provide care, and an understanding of the social and relational contexts in which they are embedded.

## Author Contributions

CP proposed the concept of family-framed care, suggested placing it within the context of the biopsychosocial, social ecological models, assumed primary responsibility for drafting the manuscript, created the figures and tables, and applied the framework to the case. SA and JP reviewed the conceptual model and affirmed that it was relevant for application to their work in Canada with caregiver-centered care, both conceptually and clinically. SA contributed to the literature review and intellectual content and reviewed and revised the conceptual framework and manuscript for clarity and consistency. JP prepared the case presentation. All authors contributed to the article and approved the submitted version.

## Conflict of Interest

The authors declare that the research was conducted in the absence of any commercial or financial relationships that could be construed as a potential conflict of interest.

## Publisher's Note

All claims expressed in this article are solely those of the authors and do not necessarily represent those of their affiliated organizations, or those of the publisher, the editors and the reviewers. Any product that may be evaluated in this article, or claim that may be made by its manufacturer, is not guaranteed or endorsed by the publisher.

## Footnotes

^1^https://www.uclahealth.org/dementia/workfiles/for-physicians/Intake-Email-8.25.2020-fillable-NEW.pdf (accessed July 17, 2021).

^2^https://www.capc.org/toolkits/implementing-best-practices-in-dementia-care/ (accessed July 17, 2021).
